# A Sustainability Compass for policy navigation to sustainable food systems

**DOI:** 10.1016/j.gfs.2021.100546

**Published:** 2021-06

**Authors:** Aniek Hebinck, Monika Zurek, Thom Achterbosch, Björn Forkman, Anneleen Kuijsten, Marijke Kuiper, Birgit Nørrung, Pieter van ’t Veer, Adrian Leip

**Affiliations:** a Environmental Change Institute, University of Oxford, United Kingdom; b Dutch Research Institute for Transitions (DRIFT), Erasmus University Rotterdam, Netherlands; c Wageningen Economic Research, Wageningen University and Research, Netherlands; d Dept. of Veterinary and Animal Sciences, University of Copenhagen, Denmark; e Division of Human Nutrition and Health, Wageningen University and Research, Netherlands; f European Commission, Joint Research Centre (JRC), Ispra, VA, Italy

**Keywords:** Sustainable food systems, Food governance, Societal goals, Food system metrics, Trade-offs, Healthy and sustainable diets

## Abstract

Growing acknowledgement that food systems require transformation, demands comprehensive sustainability assessments that can support decision-making and sustainability governance. To do so, assessment frameworks must be able to make trade-offs and synergies visible and allow for inclusive negotiation on food system outcomes relevant to diverse food system actors. This paper reviews literature and frameworks and builds on stakeholder input to present a Sustainability Compass made up of a comprehensive set of metrics for food system assessments. The Compass defines sustainability scores for four societal goals, underpinned by areas of concern. We demonstrate proof of concept of the operationalization of the approach and its metrics. The Sustainability Compass is able to generate comprehensive food system insights that enables reflexive evaluation and multi-actor negotiation for policy making.

## Highlights

• A Sustainability Compass to guide food system sustainability assessments.
• Societal goals on health, environment, equity, economy; with key areas of concern.
• Sustainability scores are calculated via a hierarchical approach to indicators.
• It enables reflexive evaluation and multi-actor negotiation of food systems outcomes.
• We demonstrate proof of concept of operationalization of the approach and metrics.

## 1 Introduction

The need for food systems transformation is broadly acknowledged (Caron et al., 2018; Fanzo, 2021; Fanzo and Davis, 2019; HLPE, 2020; Rockström et al., 2020; Webb et al., 2020; Willett et al., 2019). Design of integrated food policies is considered a key step for the navigation of food system transformation (Candel and Pereira, 2017; Galli et al., 2020). Realizing integrated policy requires evaluation of interactions between different policy goals (OECD, 2021) and consideration of diverse food system understandings (Brouwer et al., 2020). Food system sustainability frameworks are often designed to shed light on such complex dynamics in food systems and to facilitate the informed dialogue and negotiation necessary for the design of integrated food policies (Weaver and Jordan, 2008). Given the plurality of perspectives to achieving transformation in food systems (Béné et al., 2019b; Herrero et al, 2020, 2021; Klerkx and Begemann, 2020; Klerkx and Rose, 2020; Leach et al., 2020; Loboguerrero et al., 2020; Rose et al., 2021) and the growing urgency to manage trade-offs (Oliver et al., 2018; Zurek et al., 2021), it is vital that such frameworks accommodate evaluation of ‘directionality’ and ‘reflexivity’ (Kugelberg et al., 2021; TEEB 2018) to support decision making for equitable sustainability strategies (Leach et al., 2010; Patterson et al., 2017). Interest to design sustainability policies for food systems continues to grow among actors representing countries and regions (Global Alliance for the Future of Food, 2021; Global Panel, 2020; IPES-Food and ETC Group, 2021; UN Food Systems Summit, 2021). Such policy processes are best understood as dynamic and complex interactions between a diverse set of actors (from public, private, civil and academic backgrounds), each with their own knowledge and political room for manoeuvre (McGee, 2004). Sustainability frameworks that can facilitate informed dialogue and negotiation on managing trade-offs in transformation pathways for decision-makers are needed more than ever (Brouwer et al., 2020; Global Panel, 2015; OECD, 2021; Oliver et al., 2018).

A number of principles surface that sustainability frameworks must live up to in order to support policy processes. First, given the plurality of perspectives to food system transformation, frameworks should support mediation of diverse value judgements (see e.g. Lajoie-O’Malley et al., 2020; Lamine et al., 2019; Leach et al., 2020; Mockshell and Kamanda, 2018; Plumecocq et al., 2018) to seek broad societal support (Cornell et al., 2013; Reid and Rout, 2020; Saltelli et al., 2020). Second, frameworks should facilitate ‘boundary-spanning’ activities that allow for multi-actor deliberation towards a ‘shared vision’ (Termeer et al., 2018; Weber and Rohracher, 2012). Considered vital for food system assessment frameworks is the ability to provide insights on trade-offs between policy goals to decision makers (Brouwer et al., 2020; Zurek et al., 2021). Although various sustainability-oriented food system frameworks exist, they generally lack one or more important aspect of sustainability, such as equity considerations (Jones et al., 2016; Mertens et al., 2017; Stefanovic et al., 2020). Leading to the third principle: the (re-)design of sustainability strategies and food policy must build on the best possible, comprehensive evidence. These three principles are crucial for sustainability frameworks to fulfil a meaningful role in the policy process and serve as a foundation for dialogue on trade-offs and comparisons across diverse sustainability goals.

Aiming to live up to these principles, this paper presents an integrated framework that is designed to support reflexivity and evaluation of the direction of policy processes for food system transformation. Here we understand policy processes as ‘political problem-solving processes’ (Rogge and Reichardt, 2016; Weible and Sabatier, 2017) that typically involve a number of stages: ‘agenda-setting, policy formulation, decision-making, policy implementation and policy evaluation’ that are often performed in an iterative and non-linear manner (Kugelberg et al., 2021). The framework we present is designed to visualize the (lack of) progress vis-a-vis key sustainability goals and in doing so, make potential synergies or trade-offs in policy choices across different goals visible. For its operationalization it requires inputs from various models and data sources to allow for the generation of a compass to anticipate policy outcomes in a complex food system. It builds on evidence rooted in diverse sciences for a comprehensive view on food system outcomes (Cornell et al., 2013) and includes sustainability aspects that are frequently under-represented in indicator-based assessments. In the development of this framework we built on an interdisciplinary review of food system perspectives and metric-based frameworks, combined with extensive stakeholder consultation (Zurek et al., 2018). This allowed us to develop a framework that approaches sustainability in food systems from four interconnected, desired societal perspectives: *1) Healthy, adequate, and safe diets for all; 2) Clean and healthy planet; 3) Economically thriving food systems supportive of the common good; and 4) Just, ethical, and equitable food systems*. These societal goals for food systems are universal and describe desired food system outcomes. Each goal is characterized by four ‘areas of concern’. To enable the framework to accommodate concerns from all food system actors and pathways of change, selected indicators must reflect regional environmental, social, health, and economic conditions and objectives. We present an optional set of indicators, suitable for European food systems.

## 2 A review of food systems frameworks and metrics

Diverse disciplinary understandings and assessment approaches for food systems have emerged over the last decade. This is partly explained by the convergence around the notion of food systems, bringing the research focus of previously separate research communities such as agronomy, environmental sciences, nutrition sciences and agricultural economics closer together (Béné et al., 2019a; Eakin et al., 2017a; Stefanovic et al., 2020). Nevertheless, a unanimous understanding of food systems and sustainability does not exist. Definitions differ in terms of scope, boundaries, and problem statement, which together shape diverse perspectives on possible solutions (Béné et al., 2019a; Eakin et al., 2017a). Various academics have compared these diverse understandings (Béné et al., 2019a; Brouwer et al., 2020; Eakin et al., 2017a; Hospes and Brons, 2016; Stefanovic et al., 2020) and based on these, we highlight a few notable frames to understanding food systems: 1) through their value chain interactions, 2) as complex dynamic social-ecological systems, and 3) as complex webs of actors and activities that result in outcomes. More generally we see a shift towards more integrated approaches that embrace the complexity of sustainability in food systems. However, Brouwer et al. (2020) note that the relevance of existing frameworks to assessing trade-offs is limited, as they often assess value-chain sustainability. Identification of trade-offs in food systems is paramount, as they are considered a major sustainability challenge for future food systems (Zurek et al., 2021). Here, we review existing frameworks to be able to build on and complement them.

### 2.1 Existing metrics-based frameworks

The use of metrics is a firmly established tool for agri-environmental assessments (OECD, 2013; Weaver and Jordan, 2008), and increasingly so for food systems research. Metrics are perceived as useful to shed light on complex dynamics in food systems, allowing decision-makers to better navigate the system. A review of a number of metrics-based frameworks shows that all aim to offer sustainability-related insights (see Table 1). We can distinguish between metrics-based frameworks that assess the ‘status’ of food systems and those that additionally aim to offer actionable policy insights. Gradually over time, metrics-based frameworks appear to become more action-oriented and geared towards supporting policy decisions by presenting tools for stakeholder dialogue (Fanzo et al., 2020), offering intervention strategies (Willett et al., 2019), or indicating leverage points towards sustainability (Chaudhary et al., 2018). Looking across the frameworks, there is much diversity in the selection of metrics to assess food systems. We reviewed frameworks on how they addressed the following main metrics-categories: climate & environment, nutrition & health, food security, social welfare, food economy, animal welfare, food safety, and food waste. Approaches were reviewed for indicators that could be linked to these categories, allowing for comparison of these approaches.

**Table 1:** Comparison of metrics frameworks with respect to methodological and food system foci.

Paper	Year	Framework focus	Peer review	Builds on	Policy relevant output	Assessment approach	Categories of food system metrics^a^							
							Climate & environment	Nutrition & health	Food security	Social welfare	Food economy	Animal welfare	Food safety	Food waste
Sustainable Development Goals	2015	Sustainability	yes	Millennium development goals	Setting policy goals; Assessment individual goals;	17 goals that cover 169 targets, layered with 247 indicators	●	●	●	●	●		●	●
Lukas et al.	2016	Sustainable diets	yes	Systematic review literature	Nutritional footprint framework and diagram	2 metrics, layered with 4 indicators	●	●						
Jones et al.	2016	Sustainable diets	yes	Systematic review literature	Integrated metrics for LCA	30 indicators	●	●		●		●		●
Le Vallée et al.	2016	National food performance	no	OECD data comparison	Country-level report card	5 metrics, layered with between 1 and 20 indicators	●	●			●		●	
Chaudhary et al.	2018	Food system sustainability	yes	CIMSANS project	Sustainability assessment: Show trade-offs & synergies; Identify levers of change	7 food systems metrics, layered with between 2 and 6 indicators per metric, no equal weighting	●	●	●	●		●	●	●
Eme et al.	2019	Sustainable diets	yes	Systematic review literature	Integrated metrics for LCA	3 food systems metrics, layered with between 1 and 7 indicators per metric	●	●		●				
Willet et al.	2019	Planetary health diet	yes	EAT-Lancet commission	Scenarios; Sustainability intervention strategies	2 metrics, layered with between 6 indicators	●	●						●
Béné et al.	2019	Food system sustainability	yes	Systematic review literature	Sustainability assessment; Identify levers of change	4 metrics, layered with between 3 and 13 indicators	●	●	●	●	●		●	●
Fanzo et al.	2020	Food system sustainability	yes	HLPE framework	Sustainability assessment; Identify levers of change	6 metrics, layered with 4–7 indicators, based on 166 variables	●	●	●	●	●			●
Mayton et al.	2020	Sustainable diets	yes	EATS initiative	Sustainability assessment;	8 metrics, layered with between 2 and 5 indicators, based on 235 variables	●	●	●	●	●	●	●	●
Melesse et al.	2020	Food system	yes	CGIAR Food systems for healthier Diets	Integrated metrics for sustainability assessment in LMICs	9 activities and outcome metrics, layered with between 1 and 4 metrics, layered with between 1 and 8 indicators.	●	●	●	●	●	●	●	●
This paper	2020	Food system sustainability	yes	SUSFANS project	Sustainability assessment; Identify levers of change; Show trade-offs & synergies; Impact of policy interventions	4 goals, layered with 4 metrics, that are layered with between 1 and 4 indicators, based on varying numbers of variables	●	●	●	●	●	●	●	b

a The categories of food system metrics were derived through a synthesis of the reviewed papers. Scoring was based on descriptive analysis of metrics-frameworks and their mentioning of indicators that describe these categories. For illustration: *Eme et al.* score on ‘social welfare’ metrics, for their mention of ‘Income, wealth, and equity indicators’.

b The Compass does not include metrics capturing food waste. It allows to look across multiple areas of concern which enable assessment of the impacts generated by food waste (policy) interventions.

There is a clear division between frameworks that focus on sustainable diets (Eme et al., 2019; Jones et al., 2016; Mayton et al., 2020) and those that focus on sustainable food systems (Béné et al., 2019b; Chaudhary et al., 2018; Melesse et al., 2020). While the complexity of consumer food choices and diets is the starting point of sustainable diets-frameworks, the interconnectedness of multiple food systems components is a focal point for the food systems-frameworks. The sustainable diets community previously primarily leaned on nutrition, health and environmental metrics; however these increasingly incorporate a broader scope of metrics (Jones et al., 2016) and highlight issues such as acceptability and affordability (Cobiac et al., 2019; Finaret and Masters, 2019; Mertens et al., 2020). Similar trends are seen in the sustainable food systems community, where the number of indicators increases over time, as insights and knowledge on food systems develop (Béné et al., 2019b; Chaudhary et al., 2018; Gustafson et al., 2016).

While research foci differ, a multi-layered methodological approach to indicators is common; here, a metric represents a component in the food system and several indicators are then used to approach this metric. Sometimes an additional layer of metrics is used to capture overarching sustainability dimensions. Metrics for ‘nutrition & health’ and ‘climate & environment’ are most elaborate and well-defined. This is partly due to the longer traditions of indicator-based assessments for these categories, such as from the global environmental change community (IPCC, 2018) or the food security community (FAO, 2017; HLPE, 2020). Elaborate sets of metrics are presented by Willett et al. (2019), building on the ‘planetary boundaries’ (Steffen et al., 2015) and extensive research on nutritional health (Wang et al., 2019). Similarly, Chaudhary et al. (2018), show many similar environmental indicators and include indicators that cover access-related food security components. Fanzo et al. (2020) developed a ‘dashboard’ covering various food system activities, with specific emphasis on dietary health components. Finally, Melesse et al. (2020) outline a comprehensive list of potential food system-focused metrics, with specific attention to applicability in contexts with lesser quality or availability of data.

### 2.2 Several under-represented aspects

Regardless of the convergence, there are several blind spots in these frameworks. While food safety and food waste have become common for food systems frameworks, under-represented are metrics for social welfare, animal welfare, and economy (Jones et al., 2016; Stefanovic et al., 2020; TEEB 2018). Here we explore food system components that are disproportionately underrepresented (see also Table 1).

#### 2.2.1 Social justice and equity considerations

Social welfare is increasingly considered in frameworks, albeit backed by few indicators. Chaudhary et al. (2018) and Melesse et al. (2020) do so in the most extensive way (by considering gender equity, child labour, community rights, living wage), while Béné et al. (2019a) and Eme et al. (2019) define it in a narrow sense (by assessing female labour force participation or income indicators). These metrics are unable to capture social justice dynamics as described elsewhere. Exploring other literatures, a number of dynamics that need to be considered come to the fore. Firstly, literature highlights the need to consider inequitable power relations between food system actors, which in many cases perpetuate poverty and impact the already vulnerable (Clapp, 2016; Clapp et al., 2018; DeFries et al., 2017; Van der Ploeg, 2010). Such inequitable power relations stretch throughout the food system and have major effects on livelihoods worldwide (Biggs et al., 2015; Myers and Sbicca, 2015; Patel and McMichael, 2016; Thompson and Scoones, 2009). Various conditions are put forth to address or even correct these inequities: such as, providing equitable access to knowledge and technologies with respect to food system practices (Duru et al., 2015; Koohafkan et al., 2012; Wigboldus et al., 2016; Wright et al., 2016), equitable access to natural resources, such as marine (Crona et al., 2015; Daw et al., 2015) and land resources (Borras et al., 2011; Cotula, 2012); but also, the opening up of decision-making and governance processes to include voices often silenced (Duncan, 2015; Tengö et al., 2017).

The global orientation of food systems means distant places are engaged in the production of food commodities for consumers across the planet. Metrics-based approaches rarely include assessment of whether the distribution of environmental risks and benefits is just and whether there are measures in place to address these through prevention, mitigation of correction (Agyeman et al., 2004; Eakin et al, 2009, 2017b). Instead, they often make use of indicators describing the domestic situation which, for example, cannot take the potential impact of domestic food system policy on global food security into account. This requires indicators that capture how these burdens are distributed and whether they do not unfairly impact upon certain communities through ‘telecouplings’ (Eakin et al., 2009; Liu et al., 2018; Peng et al., 2019).

Moreover, IPBES shows the importance to include reflexive assessment of ‘Nature's contribution to People’ (Díaz et al., 2018): both non-material contributions, such as spiritual connections to natural landmarks (McMillan and Prosper, 2016), and material contributions, such as communities that rely on their local ecosystem (Malinga et al., 2015). So far, multi-scalar impacts of food systems related to equity considerations are difficult to monitor and assess. However, these literatures underscore several social justice and equity dimensions that urgently demand attention in metrics-based approaches.

#### 2.2.2 Animal welfare considerations

Almost entirely omitted are indicators for animal welfare (see Table 1). The lack of consideration of animal welfare reveals a contentious issue regarding the role of animal protein in food system sustainability debate (Resare Sahlin et al., 2020). On the one hand, ample work exists that argues consumption of animal protein is no longer tenable, as it is associated with persistent nitrogen-challenges (Leip et al., 2015; Poore and Nemecek, 2018; Sutton et al., 2019). Yet, on the other, academic work that underscores how animal protein continues to be essential for the dietary health and food security of many people (FAO, 2015; Hirvonen et al., 2020). Frameworks that do include the welfare of animals reared as livestock often lean on assessing the existence of legislation for animal welfare protection (See e.g. Chaudhary et al., 2018; Jones et al., 2016; Mayton et al., 2020; Melesse et al., 2020). However, research is critical of use of such variables and emphasizes there is little correlation between adherence to current requirements for more welfare-friendly production systems and an increase in animal welfare (Sherwin et al., 2010). Instead, better measures of animal welfare are outcome-based measures, which are more directly related to animal's experience (EFSA AHAW 2012). A more practical approach is assessing the impact of each of the different requirements in legislation or animal welfare initiatives and to combine this with the number of animals affected into an overall score for each species or animal group (Sandøe et al., 2020). Such metrics would better capture the debate on the role of animal protein in sustainability and would allow for trade-off analysis compared to other sustainability dimensions (Keeling et al., 2019).

#### 2.2.3 Sustainable food economy

Recent food systems literature reveals little consensus on measuring economic sustainability (Bené et al., 2019b). Indicators are often agriculture-centred and hardly capture the broader food economy and its drivers. Le Vallée and Grant's (2016) country-based ‘food report card’ leans on OECD-country ranking of economic indicators to approach ‘industry prosperity’. However, the relation to sustainability is unclear as it does not consider resource depletion and circularity. In fact, none of the reviewed frameworks capture scholarly developments that rethink how the economy can contribute to shape more sustainable food systems. For example, by exploring the notion of a welfare economy (Johansson, 1991), transformation pathways to a post-growth economy (Hinton, 2020; Koretskaya and Feola, 2020; Raworth, 2017), or sustainability pathways within the current food economy by urging existing actors to take up responsibility for sustainability, such as transnational corporations (Folke et al., 2019) or financial institutions (Loorbach et al., 2020; Ng, 2018). Nor do assessments capture other dynamics that are considered influential, such as the growing financialization of the agri-food sector and international trade. These are argued to have given way to capital accumulation and exacerbation of existing power and wealth imbalances (Schneider et al., 2020), and which might even put the ability to cultivate collective action toward sustainable food systems at risk as they exclude civil society groups and social movements (Clapp and Isakson, 2018). Moreover, the perspective on international competitiveness is frequently too narrow, leading to misguided policies and deepening inequalities within and between countries, amplifying socio-economic disparities and hampering the transition to a sustainable society (Aiginger and Rodrik, 2020).

#### 2.2.4 Food waste and food safety

While not yet universally applied, food waste and food safety are gradually acknowledged as key areas of concern, specifically in the most recent frameworks. The global-level impacts of diseases such as campylobacteriosis, salmonellosis and Avian Influenza are testament for the importance to consider food safety and zoonotic diseases linked to intensified animal production (ECDC and EFSA 2019; Leibler et al., 2009). Assessments primarily lean on data provided by the WHO, that specify the burden of foodborne illnesses (Béné et al., 2019a; Chaudhary et al., 2018). The increasing interest for food waste on the other hand, primarily sprung from the identification of its reduction as an intervention strategy for increased food security and reduced greenhouse gas emissions (Willett et al., 2019; WRI, 2018). Food waste is regarded as a loss of resources (Corrado et al., 2020; Vanham et al., 2015) that exacerbates climate and environmental impact of food (FAO, 2014; 2011; Springmann et al., 2018), and undermines efforts to reduce food insecurity (HLPE, 2014). Here, food waste is generally assessed in comparison to the total amount of food produced for a particular food group (Béné et al., 2019b; Chaudhary et al., 2018; Fanzo et al., 2020). Reduction of food waste is then considered as a key ‘environmental indicator’, while nuanced discussions on links with food safety or resilience (Bajželj et al., 2020; Mylona et al., 2018) are generally missing.

### 2.3 Metrics as boundary objects

Food system assessment frameworks have come to play a key role in informing decision-makers for the design of sustainability strategies. Nevertheless, critical reflection on the use of indicators is needed as they draw power from ‘being perceived as exact, scientific and objective’ (Lehtonen, 201576; Lehtonen et al., 2016; Sébastien et al., 2014). Lack of indicators and data for underrepresented aspects is partly explained by a persistent disciplinary-divide (Barry and Born, 2013; Weber et al., 2012), narrow interpretation of expert knowledge (Cornell et al., 2013), and a lack of transparency about assumptions and uncertainties of metrics-based assessments (Saltelli et al., 2020). In doing so, use of these frameworks in sustainability governance runs the risk of overlooking dynamics, trade-offs, or unintended consequences (Flórez Petour et al., 2018; Lehtonen, 2015). Given their ‘performativity’ vìs-a-vìs sustainability action (Hale et al., 2019), recognition and acknowledgement of biases and limitations with respect to understanding food systems is paramount. So is actively inquiring about other perspectives (Cornell et al., 2013) and transparent communication of normative values in frameworks (Saltelli et al., 2020). Only then can such metric-based understandings support transparent, inclusive, and open decision-making processes. In the following section, we present a metrics-based framework that aims for inclusion of omitted knowledges and transparency regarding choice of metrics.

## 3 The food system Sustainability Compass

A food system is sustainable when it has positive and equitable outcomes on all aspects of its environmental, social, and economic dimensions. We understand food systems as complex social-ecological systems in which networks of actors carry out diverse food system activities (production, processing, retailers, consumers, storage, disposal). These actors are driven by a wide set of driving forces and jointly produce different food system outcomes (e.g. food security and nutritional health, environmental, social and economic outcomes) (Zurek et al., 2018) and are impacted on by food system feedbacks (Ericksen, 2008; Ingram, 2011). To capture sustainability both *universally* and *comprehensively,* we have defined four societal goals to assess food system sustainability against, each of which covering four ‘areas of concern’ (see Fig. 2).

**Fig. 1:**
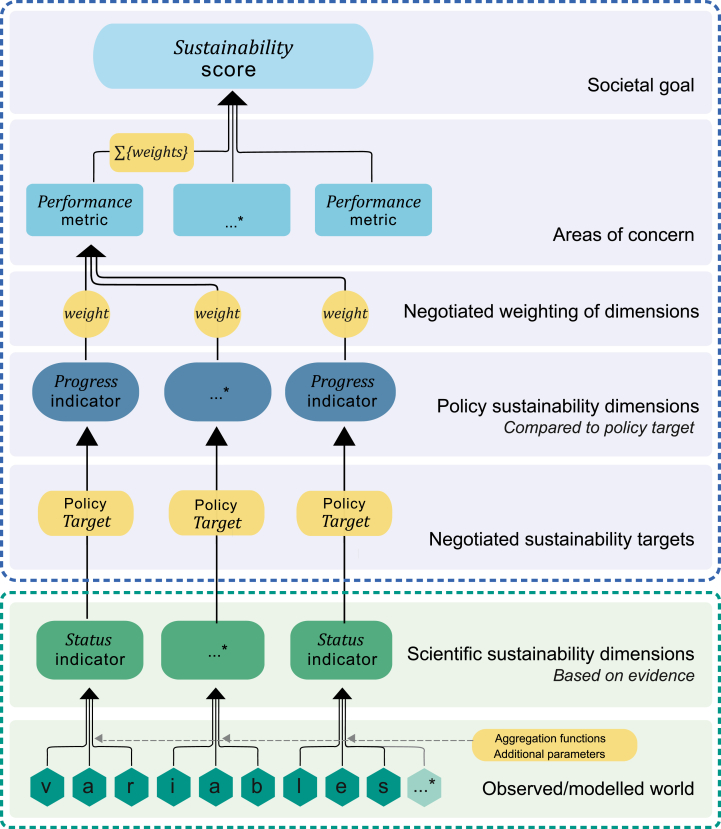
The operationalization of the Sustainability Compass through a hierarchical approach to calculate the sustainability scores.

### 3.1 Four universal societal goals for sustainability

**Healthy, adequate, and safe diets** are the basis of a healthy life and the pre-condition for successful participation in society of each individual. Delivering food security and nutritional health is the principal outcome and *raison d’être* of food systems (Béné et al., 2019a; CFS, 2012; Zurek et al., 2018). Diets of poor quality are the main contributors to the multiple burdens of malnutrition (stunting, wasting, micronutrient deficiencies, overweight, obesity, and nutrition-related non-communicable diseases) and the main cause of death (GBD 2019 Viewpoint Collaborators, 2020; Lindgren et al., 2018). Food must provide an adequate quantity of macro-nutrients (energy and protein) and micro-nutrients, but must also follow food-based dietary guidelines and contain a balanced proportion of food groups and nutrients, and sufficient food diversity (Costa Leite et al., 2020; Springmann et al., 2020). Finally, many emerging infectious diseases are believed to be zoonotic pathogens (originating from industrial animal production), whereas chemical contaminants in food contribute to long-term diseases and acute poisoning (ECDC and EFSA 2019): Diets must be safe and avoid foodborne illnesses caused by biological or chemical hazards.

**A clean and healthy planet** is the foundation for all life on earth – humans, flora and fauna. Food systems depend on natural resources and the environment, while simultaneously impacting negatively on them by extracting resources, polluting soils, air and water, and contributing to climate change and the loss of biodiversity (Rockström et al., 2020). Therefore, the elimination of these damaging impacts and the transformation of food systems to become nature- and climate-positive is elementary: in other words, regenerative food systems should not use more resources or emit more pollutants than can be replenished or absorbed sustainably, but they support restoration of biodiversity and natural landscapes. Environmental sustainability needs to be assessed at a global scale to capture the impacts along the entire food supply chain. This is done irrespectively of whether this took place inside or outside the territory for which food system assessment is performed, and across all food system spheres. Thus, quantification of indicators needs be done with suitable pressure footprints (FAO, 2018; Vanham et al., 2019; Vanham and Leip, 2020).

**Economically thriving food systems supportive of the common good** ensure livelihoods and access to food while stimulating sustainable innovation. The ultimate purpose of the economy is to support welfare in a broad sense by striking a balance between the virtues of market with the need to create and sustain common goods as well as a just society (Johansson, 1991). At its core are a vibrant and robust agri-food cluster allowing sufficient flexibility and support for individual businesses to innovate and adopt transformative practices (FOLU 2019; Herrero et al., 2020), towards emergent degrowth properties at cluster level (D'Alisa et al., 2014). Sustaining robustness on the long run requires innovative capacity to mainstream incorporation of environmental and social externalities in entrepreneurial activities (Friel et al., 2020). Robustness also requires striking a balance between self-reliance and trade to safely navigate local and global disruptions. Contributing to a just society requires fair commerce and provision of jobs that are safe, secure, and providing a living wage to all that contribute to the food system. A quality-competitive and robust food economy supportive of the common good demands balancing of the economic impacts at multiple scales (Möbius and Althammer, 2020).

**Just, ethical, and equitable food systems** are at the heart of achieving universal goals for sustainable food systems respecting of all living things. The global orientation of food supply chains results in the possibility of food system activities carried out in one location to have both positive impacts as well as unintended consequences in other, possibly distant, locations through ‘telecouplings’ (Eakin et al., 2017b; Liu et al., 2015; Wood et al., 2018), affecting humans, as well as flora and fauna (Díaz et al., 2018). Undesirable outcomes of interactions between global food trade, public health and environmental impacts are a major source of food insecurity and social and environmental injustices (Feldbaum et al., 2010; Friel et al., 2020; Kummu et al., 2020). Governing value chains towards being fair and just, demands addressing concentration of power, risk management that shifts the burden to those with less power (TEEB 2018), and the welfare of animals reared as livestock (Keeling et al., 2019; Sandøe et al., 2020). Sustainable food systems can ensure that the all living things enjoy the benefits and carry the burdens of food system dynamics in a just and equitable manner.

### 3.2 Operationalizing the Sustainability Compass

Despite the acknowledgement of, and momentum for, food system transformation, decision-makers face difficulties to design and implement the required sustainability policies for change towards desired directions. This is partly grounded in the complexity of food systems, which links both diverse policy domains, needs and value judgements, but also due the influence of policies that are already in place (Kugelberg et al., 2021; OECD, 2021; Rogge and Reichardt, 2016). Evidence-based policy making requires tools that, besides providing scientific information, can make transparent how political negotiation can or has influenced policy design (Ottersen et al., 2014). In doing so, it can illuminate weak spots in decision-making processes and highlight avenues for transparency and inclusion of diverse voices.

The food system Sustainability Compass is designed as an instrument to compare the sustainability performance of two or more states of a food system through sustainability scores. Fig. 1 shows how sustainability scores are calculated for each societal goal: scores are successively aggregated from individual variables over sustainability status and progress indicators to performance metrics, and finally to sustainability scores per societal goal (see the appendices for a glossary and the equations). This can be an ex-post evaluation, building on past development along a time series to evaluate the success of policies; or an ex-ante evaluation, anticipating results of policies on food systems based on recent insights by comparing different options or against the present situation.

**Fig. 2:**
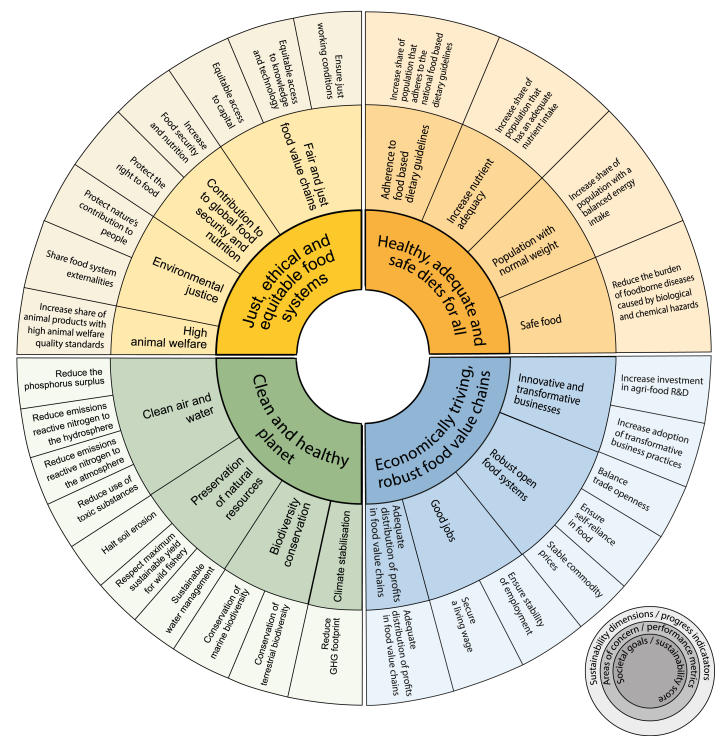
The Sustainability Compass.

To generate insights into key policy questions regarding impacts across the food system, the compass provides a framework that uses a comprehensive set of indicators together with existing policy *targets*. The use of targets allows to make progress within these areas of concern visible by showing the distance between the status of a food system from the targets. Furthermore, by showing the change in progress towards the set targets, it allows the comparison across different areas of concern, between different societal goals and between different type of metrics. This enables assessment of food system performance in relation to specific policy goals, as well as to compare performance in specific sectors. Here, we understand policy targets in the sense of ‘science-based targets' as the result of negotiation processes between actors from policy, science, and society, in contrast to ‘scientific targets’ (Rockström et al., 2021) that are primarily based on scientific insight (Rockström et al., 2021). When policy targets have not been formulated yet, we argue targets can be gleaned from broader visioning documents (e.g. the Paris Agreement). To indicate priorities amongst the different areas of concern, a set of *weighting factors* for each of sustainability dimension are included in the framework which ideally are defined through multi-actor deliberation. The simplest approach is to weigh all dimensions equally; however, it is more likely that decision-makers give higher priority to some (e.g. climate change) than others (e.g. phosphorus surplus).

Fig. 2 shows an ideal set of sustainability dimensions for assessment of EU food systems. For most dimensions, indicators and metrics have already been proposed based on existing data bases. However, this is not yet the case for sustainability dimensions that approach global impacts and animal welfare. To operationalize the compass, those gaps need to be addressed in pragmatic, yet relevant ways. We show proof of concept with Table 2, which exemplarily demonstrates *one* sustainability dimension per area of concern from the full set proposed in Fig. 2. We follow (Béné et al., 2019b) in making selection criteria for indicators transparent, as they provide valuable insights in how the framework approaches assessment of food systems. In the development of indicators, the following set of criteria was prioritized:

• *Pragmatic*: pragmatic solutions (e.g. use of proxies) are necessary when data is not available or accessible.
• *Unique*: redundancy and double counting of variables are avoided.
• *Relevant*: indicators need to capture the essence of the problem rather than be guided by data availability or previous existence of indicators; in some cases, composite indicators are necessary.

**Table 2:** Proof of concept for the operationalization of the sustainability compass.

	Area of concern	One of the sustainability dimensions	Example for representative Status Indicators	Possible database	(Potential) target	Note
Healthy, adequate and safe diets for all	Adherence to food based dietary guidelines	Increase share of population that adheres to the national food based dietary guidelines	Share of citizen with dietary patterns that are compatible with national Food Based Dietary Guidelines (FBDGs).	EFSA Comprehensive Food Consumption Database FAO FBS	*Reduction of people not adhering to FBDG/healthy diet indices to x%.* ***SDG 2.1 target*** *by 2030 end hunger and ensure access by all people, in particular the poor and people in vulnerable situations including infants, to safe, nutritious, and sufficient food all year round.*	Food based dietary guidelines (FBDGs) provide advice on consumption of particular food groups and dietary patterns to promote overall health and prevent chronic diseases, adapted to the national context (Herforth et al., 2019; Wijesinha-Bettoni et al., 2021). A growing body of evidence points out that specific foods and dietary patterns have a substantial role in the prevention of chronic diseases, such as cardiovascular diseases, certain cancers, and type 2 diabetes (Wang et al., 2019), though some FBDGs are evaluated incompatible with the agenda on non-communicable diseases (Springmann et al., 2020) and do not address all dietary pattern across the countries (Costa Leite et al., 2020). As FBDGs often don't provide quantitative data, indices such as the ‘Healthy Eating Index’ (Guenther et al., 2013) or ‘Alternative Health Eating Index’ (Chiuve et al., 2012) have been developed. As a first approach, national food consumption data can be used, but due to the variability in food consumption, and different needs for sub-population groups, the use of disaggregated national food surveys should be used if available (Mertens et al., 2018).
	Increase nutrient adequacy	Increase share of population that has an adequate nutrient intake	Prevalence of nutritional deficiencies as cause for non-communicable disease	GHDx Global Burden of Disease USDA nutrient composition data	*Reduction of occurrences of NCD caused by nutritional deficiencies to x%* ***SDG 2.2 target*** *By 2030, end all forms of malnutrition, including achieving, by 2025, the internationally agreed targets on stunting and wasting in children under 5 years of age, and address the nutritional needs of adolescent girls, pregnant and lactating women and older persons.*	Various indicators are used to calculate Food Nutrition Adequacy (Chaudhary et al., 2018; Melesse et al., 2020). The Global Burden of Disease ‘Global Health Data Exchange’ provides data on deaths and disability-adjusted life years caused by nutritional deficiencies (protein, iodine, vitamin A, iron, other) (Global Burden of Disease Collaborative Network, 2017). More detailed Nutrient Rich Diet (NRD) scores are e.g. NRD9.3 (Drewnowski, 2009; Fulgoni et al., 2009) or other scores based on a higher number of nutrients (Drewnowski et al., 2021; Grigoriadis et al., 2021; Mertens et al., 2018). Such scores can be calculated if detailed food consumption data are available from food balance sheets or food surveys, in combination with food nutrition databases.
	Population with normal weight	Increase share of population with a balanced energy intake	Prevalence of obesity, e.g. BMI≥30 [% of adult population]; Child and adolescent overweight (BMI-for-age > 1 SD)	NCD Risk Factor Collaborators	*Reduction of obesity to x%* ***WHO target*** *At the 2013 assembly, member States agreed to a set of voluntary targets to reduce NCDs, including to, by 2025, halt the rise in obesity at 2010 levels.* ***SDG 2.1 target****(see above)* ***SDG 2.2 target****(see above)*	Recent estimates suggest that globally nearly 690 million people are hungry, 144 million children under 5 years of age stunted and 47 million wasted (FAO, 2018). Overweight and obesity is a growing not only in high-income countries but also in lower-middle and low-income countries, affecting globally 13% of obese adults in 2016, with peak values over 30% in some countries (FAO, 2018). While all forms of malnutrition need to be addressed in the food system sustainability compass, we present here a possible indicator addressing obesity. Indicators on food security are generally well developed and available in data bases (e.g. SDG Tracker, https://sdg-tracker.org/zero-hunger; Food Systems Dashboard https://foodsystemsdashboard.org/). Balanced energy intake is a function of demography, body metrics, and activity levels (Bodirsky et al., 2020; van den Bos Verma et al., 2020) and disaggregated scores can be calculated are if detailed consumption data are available.
	Safe food	Reduce the burden of foodborne diseases caused by biological hazards	Burden of foodborne illness (number of cases)	ECDC Surveillance Atlas of Infectious Diseases WHO Foodborne disease burden database (soon available)	*Reduce cases of foodborne illnesses by x%* ***WHO target*** *Lowering the burden of foodborne diseases.*	Foodborne illnesses are most often infectious or toxic by nature and caused by bacteria, viruses, parasites or chemical substances entering the body through contaminated food or water and can cause mild or severe diarrhoea or systemic infections (ECDC (European Centre for Disease Prevention and Control) and EFSA (European Food Safety Authority), 2019). Occurrence of antimicrobial resistance in zoonotic and indicator bacteria from humans, animals and food is of growing concern (Jansen et al., 2019; Tacconelli et al., 2018). Targets are set for prevalence and or numbers of specific pathogenic microorganisms in foods and food producing animals, but these are not necessarily directly linked to number of human cases. No target in relation to human disease is articulated but one potential target could be the acceptable number of recorded cases of a specific foodborne disease (i.e. salmonellosis and others).
Economically thriving food systems supportive of the common good	Innovative and transformative businesses	Increase adoption of transformative business practices	Companies publishing sustainability reports.	SDD - GRI Database Carrots & Sticks - Reporting instruments	*Number of companies publishing sustainability reports* ***SDG 12.6 target****: encourage companies, especially large and trans- national companies, to adopt sustainable practices and to integrate sustainability information into their reporting cycle*	The role of the private sector is crucial for sustainability transformation (Folke et al., 2019; FOLU et al., 2019; United Nations Environment Programme, 2021). Promising new business models and practices that support sustainability emerge in niches across food systems (Kuokkanen et al., 2019), building nature-positive economy principles. For conventional businesses, sustainability principles are increasingly becoming a standard evaluation criteria for businesses (Escrig-Olmedo et al., 2019). It is vital they make transparent how they address sustainability in their business practices so they can be held accountable (Folke et al., 2019). While ESG-reporting is becoming common practice, uniform requirements for reporting are lacking and it cannot compare to the rigour of e.g. financial reporting (Galvin et al., 2014; Halbritter, 2015). While data that captures the impact of the private sector on sustainability thus far are lacking, the number of companies that publish sustainability reporting provides opportunity to assess corporate sustainability policies. In fact, reporting on sustainability is increasingly made mandatory on the national level, pushing transparency from the private sector on their social and environment impacts (Van der Lugt et al., 2020). The GRI database shows the number of companies that have voluntarily registered they publish sustainability reports. Other indicators could be the number of national laws that make sustainability reporting national, as captured by Carrots & Sticks, or the use of private certification schemes, such as B Corp as suggested by (Folke et al. (2019).
	Robust open food systems	Stable commodity prices	Price volatility index	FAOstat monthly CPI	*Target* = *stay below a maximum fluctuation*	Extreme food price volatility led to the food crisis in 2007–2008, which suddenly pushed into food insecurity (Patel and McMichael, 2016). These events have highlighted the entanglement of financial speculation and the stability of food systems (Clapp and Helleiner, 2012). Stable food prices are considered vital to ensuring access to sufficient, healthy food, specifically in low-income countries where household can spend as much as 75% of their income on food (Global Panel, 2016). To assess the stability of food commodities, we propose the indicator suggested by Béné et al. (2019b).
	Good jobs	Secure a living wage	Ratio of income share held by highest 20% to the lowest 20%	Food System Dashboard	*Reduction of income ratio by x%.* ***ILO target:*** *‘Decent work for all’ is the organizing framework and principal objective of the ILO since 1999* ***UN target*** *2030 Agenda for Sustainable development took integrated the ILO target*	Income is generally considered a vital indicator to assess whether sectors are able to provide good jobs in order to combat poverty and inequality. While many nations have data on minimum wages, such indicators cannot capture whether work is in fact good (Anker and Anker, 2017). To assess whether wages are fairly distributed across the food system, we assess the gap between the income of those that receive the highest salary and those that receive the lowest is. An alternative indicator is to capture the % of people receiving living wage. In order to capture whether work is able to ensure an income that can uphold a basic, yet decent standard of living for a worker and their family, the notion of ‘living wage’ was defined (Anker and Anker, 2017). Living wages differ between countries, and sometimes even between regions within countries as costs of living might be lower in rural areas than in urban areas (globallivingwage.org).
	Adequate distribution of profits in food value chains	Adequate distribution of profits in food value chains	Farm gate prices Primary value-added versus food processing value added	Word Development Indicators	*Increase share of added value belonging to agriculture and fisheries in the total food supply chain by x%.*	Unequal bargaining power balances in the food supply chain might lead to unfair trading practices leaving little room to negotiate contracts by the weak party, which – in a globalized food system – often are the primary producers vis-à-vis internationally acting food processing and retain companies (Fałkowski et al., 2017; Group of Chief Scientific Advisors, 2020; Leip et al., 2021). Amongst the results, reduced farm gate prices with consequences of farm and workers' income are discussed (Marcantonio et al., 2018), but also risks of over-production and increased food waste, decreased investments in innovation and technology (European Parliament, 2012). While the share of businesses is largest in the farming sector, the share of value added of agriculture was in Europe only 25% of the total food chain value added in the years 2008–2014 (European Commission, 2017). The World Development indicators give data with global coverage on added value in agriculture, forestry and fishery, food and beverage industry and in the manufacturing sector.
Clean and healthy planet	Climate stabilisation	Reduce GHG footprint	Sum of domestic GHG emissions from food systems and embodied emissions in trade	EDGAR-FOOD GHG inventories FAOSTAT Food Balance Sheet	*Food system GHG reduction targets are not generally defined but could be aligned with general national GHG reduction target according to national legislation or the National Determined Contributions.* ***EU Green Deal target*** *Cut GHG emissions 55% by 2030 – compared with the ambition of a 40% cut of 1990 levels. By 2050, the EU should reach net-zero carbon emissions, compared to the previous goal of an 80% reduction.*	Environmental indicators need to capture both the territorial aspects, as well as the embodied environmental effect in trade. Most sustainability framework (see Table 1) include databases measuring progress towards environmental objectives capture the territorial aspects only. As suggested by Sala et al. (2020), a combination of these data sets - where available - with 'footprints' for traded goods captures the ‘systemic’ effect of footprints. We propose here a combination of activity-based emission accounting (consistent with national GHG inventories) and footprints. GHG inventories cover the ‘territorial’ part of GHG emissions. This needs to be complemented with embodied GHG emissions in imports, which could be calculated using commodity trade flows (e.g. FAOSTAT Food Balance Sheets) combined with LCA factors (Poore and Nemecek, 2018).
	Biodiversity conservation	Conservation of marine biodiversity	Reduce use of plastics and ecosystem disturbances through fisheries: Reduce capture fisheries	Our world in data Seafood production (original data: World Bank – World Development Indicators)	*Objective should be the ban of unsustainable fisheries. If data on ‘unsustainable fisheries' are missing a reduction target needs to be agreed.*	Marine biodiversity is endangered by the presence of toxic substances (with plastics being in the focus of recent discussions), disturbances by unsustainable fishery practices (e.g. trailing gears) affecting target and non-target species as well as ecosystems, and by alteration of its chemical status, particularly reduction of the pH leading to coral bleaching (Grip and Blomqvist, 2020; Price, 2001; Wang et al., 2021). While the chemical effect is captured already by emissions of GHGs and Nr, we suggest the size of fisheries as a proxy for emissions of plastics and disturbances, using ‘total’ fisheries in the absence of better data on ‘sustainable fisheries'. Plastics per se are inert, but often contain toxic substances that can bio-accumulate in the marine food chain. Food system land-sourced plastics are (yet) difficult to quantify, amongst many other sources (e.g. cosmetic, with microplastics directly entering the hydrosphere, while macroplastics (e.g. in packaging) might be unproblematic for the marine environment if properly managed (Simul Bhuyan et al., 2021).
	Preservation of natural resources	Sustainable water management	Reduce the blue and green water footprint	FAO FBS Water footprint network	*Reduce blur and green water footprint by x%.* ***SDG target 6.4*** *By 2030, substantially increase water-use efficiency across all sectors and ensure sustainable withdrawals and supply of freshwater to address water scarcity and substantially reduce the number of people suffering from water scarcity.*	Both water stress/scarcity and water footprints are relevant for sustainability (Vanham and Leip, 2020); SDG 6.4.2 is available from AQUASTAT; as additional indicator, water footprint can be calculated based on the data from the Water Footprint Network (Mekonnen and Hoekstra, 2011, 2012) and the FAO FBS.
	Clean air and water	Reduce emissions of air pollutants to the atmosphere	Reduce domestic emissions of reactive nitrogen to the atmosphere and b) reduce embodied Nr emissions in trade: feed imports	CLRTAP reported emissions from the EMPE Centre of Emission Inventories and Projections available for UN-ECE countries, all air pollutants. UNFCCC reported emissions of NOx and SO2 from energy-related sources, as well as NH3+NOx emissions from agricultural sources are available in CRF tables	*Reduce Nr footprint by x%* ***UNECE target:*** *Emissions ceilings of air pollutants for UN-ECE are defined under the Gothenburg protocol (Annex II);*for countries of the European Unition in the NEC Directive ***EU Green Deal target*** *Composite targets of reduction of reactive nitrogen emissions by 50% have been defined in the Colombo Declaration and the EU Farm to Fork Strategy*	Air pollutant emissions can be calculated from national GHG or air pollutant inventories; possibly considering also non-agricultural sectors contributing to food system emissions according to Crippa et al. (2021). We are aware that the indicators proposed do not cover all potential impacts that are e.g. covered by LCA guidelines. The ReCiPe method differentiates 17 midpoint categories with three endpoint areas of protection (damage to human health, damage to ecosystems, damage to resource availability) (RIVM, 2017). While LCA is mostly applied at the individual supply chain level, applications to the ‘macro’ level have already been successful by using a ‘Basket of Product’ approach (Castellani et al., 2017) or by combining LCA with input-output models (Beylot et al., 2019).
Just, ethical, and equitable food systems	High animal welfare	Increase share of animal products with high animal welfare quality standards	Share of certified organic products sold.	FiBL database	*Increase share of certified organic product by x%* ***EU Green Deal target*** *The EU Farm to Fork and Biodiversity Strategies have set a target of 25% of area under organic farming to increase organic agriculture and aquaculture by 2030 aiming at both environmental and animal welfare objectives.*	Animal welfare is an important concern for EU citizens and consumers with 94% believing it to be important and 84% believing that it needs to be improved (EU-barometer, 2015). However, despite progress in understanding animals' needs and a general acceptance of the principles of the Five freedoms for defining animal welfare, no (internationally agreed) indicator or database exists that could be used as a basis for this area of concern (Sandøe et al., 2020). Animal welfare labels have been developed but are generally only used on a national level (Beter Leven, 2021; RSPCA, 2021). Organic standards generally have stricter animal standards than conventional agriculture which may improve a number of aspects of animal welfare (Duval et al., 2020; Van de Weerd et al., 2009). Also, specific production system (e.g. jamon iberico) are subject to regulations perceived to be consistent with higher animal welfare standards (García-gudiño et al., 2021). Therefore - until more direct indicators and data bases are developed - we suggest using the share of organic and animal welfare certified animal products.
	Environmental justice	Share food system externalities	Green financing: budget spent	*Data are usually confidential, thus no publicly available database was identified/*	*Increase budget on green financing by/to x%.*	How to define a ‘fair share of environmental externalities' is a difficult and controversially discussed issue (Morin et al., 2018). Most advanced is this discussion in the area of ‘climate justice’ (Seyfang and Paavola, 2008) proposing e.g. an equal share of the remaining ‘carbon budget’ on a per-capita basis, with or without accounting of the historic emission paths (Acar et al., 2018). Countries consuming higher shares endanger the global (common) objective of keeping the earth within its ‘safe and just’ corridor (Rockström et al., 2021), but are also contributing to the impacts outside their own countries. While the first issue needs to be captured in areas of concern under the ‘Environment’ social goal (considering full life-cycle footprints), the second issue must be captured to ensure ‘just, ethical and equitable food systems'. As a possible indicator we suggest here the budget spent on Green or Climate financing mechanisms such as the Global Environment Facility (GEF) Trust Fund or the Adaptation Fund. (Khan et al., 2020). Further possible indicators could include the ‘biocapacity’ (reserve/deficit) based on the methodology of the Global Footprint initiative (Lin et al., 2018; Mancini et al., 2018; Świader et al., 2018), with the target to reduce the global footprint below 1 where there country's ecological footprint doesn't exceed its biocapacity.
	Contribution to global food security and nutrition	Increase global food security and nutrition	Impact on GFSI: GFSI overall score for countries with a score below a threshold in the reference situation, weighted by trade flows with the partner countries.	Global Food Security Index	*Increase of the “Impact on GFSI” by [x%] or [y points]' or [z point/year]*	Trade is an important source of income for contributing to the development of developing countries (Kummu et al., 2020). On the other hand, trade flows can also undermine local economies therefore decreasing welfare and thus food security in developing countries (Wood et al., 2018). Sustainable food systems support the first and avoid the second effect. For example, the European Union adopts the Generalised Scheme of Preferences (GSP, European Union, 2012) that should help developing countries to alleviate poverty and create jobs with partially or fully removed duties, bound to the condition that beneficiary countries respect the principles of fifteen core conventions on human rights and labour rights. Further possible indicators could include ‘Increase of spending contributing to projects and/or international institutions (e.g. WFP) working on the reduction of food insecurity in vulnerable countries.
	Fair and just value chains	Ensure just working conditions	Impact on ILO scores: ILO scores for countries with a score below a threshold in the reference situation, weighted by trade flows with the partner countries.	ILO database on Safety and health at work, working poor, labour income and social protection	*Increase of the “Impact on ILO scores” by [x%] or [y points]' or [z point/year]*	Trade is an important source of income for contributing to the development of developing countries (Kummu et al., 2020). On the other hand, irresponsible supply chains might also contribute to undermining working conditions and workers' rights in trading partner countries seeking to enhance competitiveness on the market (Harrison et al., 2019). Trade agreements could be an instrument to enforce regulations that ensure workers' rights, safe working conditions, and fair wages, as laid out in the eight fundamental Conventions by the International Labour Organization (ILO) defining standards on the freedom from forced labour (Convention 29 and 105), the freedom to form and join a union and to bargain collectively (Convention 87 and 98), the freedom from discrimination at work (Convention 100 and 111) and the freedom from child labour (Convention 138 and 182). Bilateral trade agreements more and more include a so-called specific chapter on Trade and Sustainable Development (TSD) that links to the enforcement of the ILO conventions, possibly including also further conventions on the monitoring of the labour conditions (Harrison et al., 2019). For example, the recent bilateral trade agreement between the EU and Vietnam includes a detailed TSD on labour, biodiversity, climate and other sustainability aspects. Article 13.13 of the agreement (EU, 2018) formulates that “*The Parties shall, jointly or individually, review, monitor and assess the impact of the implementation of this Agreement on sustainable development through their respective policies, practices, participative processes and institutions.*" Further possible indicators could include e.g. ‘Share of products certified with high social standards (e.g. Fairtrade label, Rainforest Alliance)".

These criteria differ from those of other indicator frameworks putting more emphasis on data availability or global applicability. The selection of examples (Table 2) captures the range of challenges that are faced both conceptually and with regards to data.

## 4 Policy implications

In order to support decision-making, the Sustainability Compass needs to be adapted to the specific context of the food system of interest. The framework supports this process by indicating what decision-makers should reflect on and negotiate with stakeholders: the selection of appropriate sustainability dimensions and indicators for each area of concern, and the definition of targets and weighing factors. Secondly, the compass is designed such that it can be used throughout the policy process to ensure *reflexive and comprehensive evaluations* of food systems, but also to foster *multi-actor and inclusive negotiations* on food systems (see Table 3).

**Table 3:**
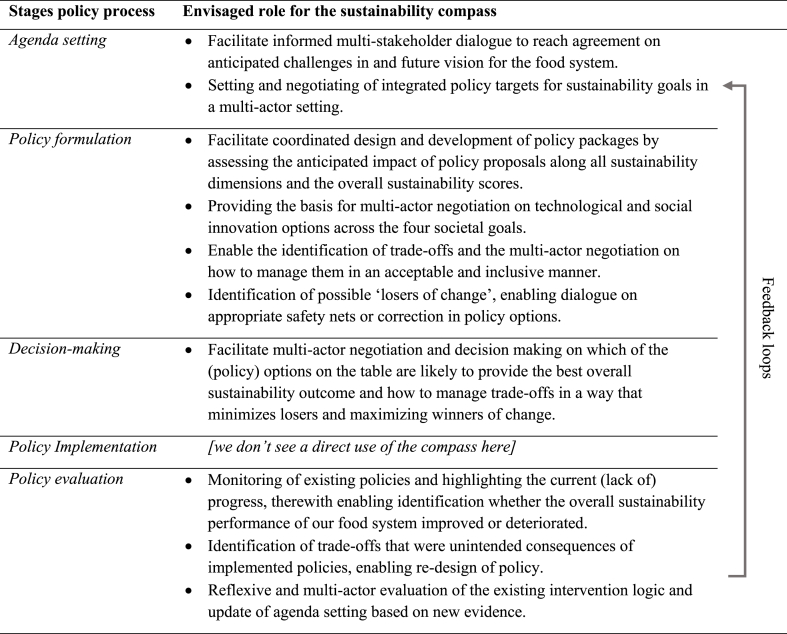
Use of the Sustainability compass in major stages of policy process as set out by Kugelberg et al. (2021).

Central to the compass is the ability to facilitate multi-actor negotiations on food system sustainability by allowing diverse stakeholders to make sense of the complex adaptive nature of food systems. We argue that frameworks that can show a comprehensive, yet comprehendible picture of the food system is key to facilitate such informed multi-actor dialogue and negotiation. The presented compass can support the process of fine-tuning the multi-dimensional direction of travel towards a food system vision, which in turn enables decision makers to re-adjust targets and weighting for diverse sustainability dimensions. Moreover, it can help evaluate discrepancies between scientific targets and negotiated, often more modest, science-based targets that have been set (Rockström et al., 2021), therewith giving an indication of the ambition for change of targets. As such, the conceptual framework (even without data) can support dialogue on what is desired change and what are acceptable trade-offs.

Second, the Sustainability Compass enables reflexive evaluation when it is used in combination with data. In this way, it can make the observed or anticipated impact of a policy package visible for evaluation and therewith enabling reflexivity to fine-tune policy proposals. For this, the Compass leans on its ability to make visible the distance-to-target, therewith providing a tool to standardize the impact assessment of policies. Moreover, by showing impacts across a comprehensive set of areas of concern, it can highlight possible trade-offs and synergies between the four societal goals - or amongst their areas of concern. The design of the framework is flexible to incorporate newly generated insights on scientific evidence or negotiated sustainability dimensions of weighting. A further development to increase its policy use, is to enable the compass to simulate or visualize how the introduction of different innovations (Herrero et al., 2021; Klerkx and Begemann, 2020; Zurek et al., 2021) can change the states of areas of concern, thus allowing a comparison between (possible) effects the directionality of change of diverse innovation options.

The recognition that ‘better regulation’ is urgently needed to address sustainability challenges is growing. In fact, an increasing number of institutions emphasize to strive for more transparent, inclusive, and evidence-based policymaking (EC 2015; European Union, 2016). With the Sustainability Compass we provide a comprehensive framework with the intention to fully cover a wide range of possible stakeholders' interests through its areas of concern in a way that can be contextualized and specified through the choice of sustainability dimensions. Areas of concern are proposed as being ‘universal’ but reflect what we reviewed as current interpretation of sustainability: this is not constant over time. Our proposal of embedding the Sustainability Compass into the policy process aims at 1) transparent interplay between scientific evidence driven and normative, value driven decisions for food system assessment ensuring overall evidence-based decisions; and 2) to mirror the process of the UN Sustainable Development Goals and separate the process of development of sustainability indicators and their use in policy assessment.

## 5 Conclusion

Transformation of food systems is urgently needed; failing to do so is frankly not an option. We define food systems as sustainable when they are able to provide healthy, adequate, affordable and safe diets, which are the basis of a healthy life and the pre-condition for successful participation in society of each individual, while safeguarding a clean and healthy planet recognizing it as the foundation for all life on earth. To achieve this, sustainable food systems are economically thriving and supportive of the common good, in the pursue of universal goals in a just, ethical, and equitable manner. With the Sustainability Compass we contribute a comprehensive sustainability assessment framework that can enable an inclusive and transparent policy dialogue and provide actionable insights. The compass can be used throughout the various stages of policy processes to foster multi-actor, inclusive negotiations and ensure reflexive and comprehensive evaluations. In doing so, it provides the foundation for the design of integrated policies that reflexively manage trade-offs. While implementation of the framework hinges on availability of data and consensus on policy targets, these can be overcome on the short term with pragmatic solutions as presented in this paper.

## Disclaimer

The views expressed in this paper are those of the authors and do not necessarily reflect the position of the European Commissions.

## Declaration of competing interest

The authors declare that they have no known competing financial interests or personal relationships that could have appeared to influence the work reported in this paper.

## Appendix 1 Glossary

**Food systems** are social-ecological systems in which networks of actors carry out diverse food system activities (production, processing, retailers, consumers, storage, disposal), driven by a wide set of driving forces and jointly producing different food system outcomes (e.g. food security, environmental, social and economic outcomes).

**Sustainable food systems** are systems that have positive and equitable outcomes on all aspects of its environmental, social, and economic dimensions. What this entails for each of the dimensions is captured in societal goals.

**Societal goals** are universal societal goals that describe the desired functionality of food systems.

**Sustainability scores** combine performance metrics to give a comprehensive sustainability assessment for a societal goal.

**Areas of concern** capture what are main areas of concern for reaching a societal goal. In other words, meeting a societal goal requires addressing all areas of concern.

**Performance metrics** combine one or several progress indicators into one metric to describe the performance in an area of concern that is deemed important to reach a societal sustainability goal.

**Sustainability dimensions** are used to approach areas of concern and can provide insights to policy progress made or the status of system.

**Progress indicators** transfer status indicators from the scientific domain to the policy domain by comparing the ‘status’ of the sustainability dimension against a goal, such as a policy target or vision.

**Status indicators** combine several individual variables to make up a new measure for the status of a sustainability dimension of the food system (e.g., nitrogen input vs. output, ratio of energy intake vs. expenditure). Sometimes additional information is required to derive the variable (e.g., conversion of individual Greenhouse Gas Emissions to total CO2 equivalents). The definition of a status indicator aligns with the scientific definition of a sustainability target or vision that allows assessing progress.

**Variables** are measures that can be quantified and/or counted in commonly used standards (e.g., hectares, kg).

**Policy targets** are negotiated targets for sustainability progress which are the result of policy processes. These a negotiation between politics, science, and might have been influenced by lobby groups. Policy targets are partially related to scientific evidence but have been adjusted by stronger value judgements and political limitations.

**Visions** are accepted aspirational goals, such as the 2 °C Paris target or the Sustainable Development Goals. Visions are generally primarily evidence-based with ‘some’ negotiation.

## Appendix 2 Equations for operationalization of the Sustainability Compass

The assessment of a policy package starts with the quantification of the indicators to describe the ‘status’ in a reference (*Status A*) and a comparison situation (*Status B*) (e.g. presence or business as usual scenario vs. policy scenario, etc.). Assessing whether or not the policy package improves food system sustainability then requires comparing the change from *Status A* to the future *Status B* with the intended change based on the pre-defined targets, thus translating *Status indicators* to *Progress indicators*. Hence, the *Progress indicator* quantifies the relative distance to the set target that has already been achieved (see Equation (1)).

(1) $$\mathrm{Progres}s_{i_{p}}=\frac{\mathrm{Statu}s_{i_{p}}^{B}-\mathrm{Statu}s_{i_{p}}^{A}}{\mathrm{Targe}t_{i_{p}}-\mathrm{Statu}s_{i_{p}}^{A}}$$

with $i_{p}$ the number of sustainability dimensions used for the area of concern *p*.

The *Progress indicator* is dimensionless, meaning its calculated value usually ranges between zero and one. However, it can also be larger than one (‘overshooting’) or negative (‘deterioration’). *Performance metrics* (*P*) are calculated as the weighted average of the progress indicators (Equation (2)). They can subsequently be combined to sustainability scores for each societal goal (Equation (3)):

(2) $$\mathrm{Performanc}e_{p}=\frac{\sum_{i_{p}}\left\{\mathrm{Progres}s_{\mathrm{ip}}\cdot \mathrm{weigh}t_{i_{p}}\right\}}{i_{p}}$$

With *p* the number of areas of concern for the respective societal goal.

(3) $$\;\mathrm{Sustainabilty}=\frac{\sum_{p}\left\{\mathrm{Performanc}e_{p}\cdot \sum_{i_{p}}\left\{\mathrm{weigh}t_{i_{p}}\right\}\;\right\}}{\sum_{p}\sum_{i_{p}}\left\{\mathrm{weigh}t_{i_{p}}\right\}}$$
